# Isolation of different fungi from the skin of patients with seborrheic dermatitis 

**DOI:** 10.18502/CMM.6.2.2841

**Published:** 2020-06

**Authors:** Elaheh Mahmoudi, Jahangir Rezaie

**Affiliations:** 1 Department of Mycology, School of Medicine, Alborz University of Medical Sciences, Karaj, Iran; 2 Department of Medical Laboratory Sciences, School of Paramedicine, Alborz University of Medical Sciences, Karaj, Iran

**Keywords:** *Malassezia*, Non-*Malassezia*, Seborrheic dermatitis

## Abstract

**Background and Purpose:**

Seborrheic dermatitis (SD) is characterized by erythematous inflammatory patches that mostly appear in the sebaceous gland-rich skin areas. In addition to the key role of *Malassezia* species in SD, its contribution to other fungal microbiota has been recently addressed in the literature. Regarding this, the present study was conducted to identify and determine the fungal species associated with the incidence of SD.

**Materials and Methods:**

For the purpose of the study, fungal microbiome in scaling samples were collected from SD lesions and then analyzed based on the DNA sequencing of ITS regions.

**Results:**

In addition to *Malassezia*, several fungal species were detected in the samples collected from the SD lesions. According to the results, 15.5%, 13.3%, and 6.7% of the isolates were identified as *Candida parapsilosis, Cryptococcus albidus var. albidus/ Rhodotorula mucilaginosa,* and *Penicillium polonicum*, respectively.

**Conclusion:**

Based on the obtained results, *C. parapsilosis* was the most prevalent non-*Malassezia* species isolated from SD lesions. Our results provided basic information about a specific fungal population accounting for the incidence of SD.

## Introduction

The human skin mycobiome is comprised of a variety of fungal species [ 1 - 4 ]. Fungal microbiota reportedly undergoes a change with the incidence or severity of certain skin diseases [ 5 - 8 ]. For example, the number of *Malassezia* yeasts is increased 1.5-2 times, compared to the normal levels, on the seborrheic dermatitis (SD)-afflicted crust [ 7 , 8 ]. The SD is characterized by erythematous patches that mostly appear in sebaceous gland-rich areas in the scalp, forehead, eyelids, nasolabial folds, and upper trunk [ 9 ]. 

*Malassezia* lipophilic yeasts play a key role in the development of SD; however, its contribution to other fungal microbiota for the recurrence and severity of the disease has been addressed in the literature [ 10 - 11 ]. Simultaneous presence of* Candida* species and *M. pachydermatis* in canine SD lesions is associated with the exacerbation of the clinical symptoms of SD. The co-colonization of these two yeasts is related to the formation of a much higher amount of extracellular polymeric substance matrix (biofilm). This matrix protects microbial cells against adverse conditions and causes chronic infections by tightly attaching them to the surface material [ 12 ]. Determination of the associations among *Malassezia* species or between these species and other fungi can facilitate the establishment of therapeutic procedures for SD. Regarding this, the present study was conducted to comprehensively analyze the skin fungal microbiota in SD lesions.

## Materials and Methods

***Patients and specimens***

The Ethics Committee of Alborz University of Medical Sciences, Karaj, Iran, approved all experiments conducted in the study with the code of Abzums.Rec.1395.51. This study was conducted on 19 patients referring to the Skin Department of Bahonar Hospital, Karaj, Iran. The lesions with yellowish scale, pityriasiform scaling, and greasy seborrhea and erythematous plaques were clinically diagnosed as SD [ 13 ]. The patients who had used antifungal drugs or other medications, such as anti-inflammatory and antimicrobial drugs, during the previous 6 weeks [ 14 ], were excluded from the study.

***Isolating and identification of the fungus inducing seborrheic dermatitis***

Microscopic identification was performed on the collected samples using the direct KOH test on a wet mount. Skin samples collected from the SD lesions were inoculated on modified Dixon’s agar (Sigma, Germany), supplemented with cyclohexamide (0.5%), chloramphenicol (0.05%), and Sabaro dextrose agar (Sigma, Germany). Subsequently, the samples were incubated for 10 days at 32°C for the isolation of *Malassezia* and for 3-5 days at 37°C and 24°C for the isolation of other fungal species. The obtained colonies were identified based on the morphological characteristics of the colonies, attendance of catalase, germ tube examination [ 15 ], and Tween assimilation test [ 8 ].

**Molecular identification **

Polymerase chain reaction (PCR) was performed by isolating the fungal genomic DNA using the phenol-chloroform protocol described by Yamada et al. [ 16 ]. The PCR amplification of genomic DNA was carried out to amplify the ITS-5.8S rDNA region using the universal primers ITS4 and ITS5 [ 17 ]. The PCR was performed in 25 µl of a 2-µl Taq 2x PCR Master Mix (SinaClon BioScience Co., Karaj, Iran), 0.5 µl of each primer, and 2 µl DNA templates using a PCR thermal cycler (Peqlab, Belgium). The thermal cycle consisted of denaturation for 5 min at 94°C, 35 cycles of a second denaturation at 94°C for 45 sec, annealing at 56°C for 40 sec, and elongation at 72°C for 2 min. The PCR was completed through a final elongation at 72°C for 10 min. 

Further gel extraction of the PCR products and sequencing were performed using the Biosystems 3730 XL Bioneer DNA analyzers (Korea). The molecular database recorded at NCBI Medical Library, Bethesda, MD, USA (http://www.ncbi.nlm.nih.gov/BLAST/) was also employed for the identification of the isolated fungal species.

**Statistical analysis**

The data were analyzed using SPSS software, version 15 (SPSS Inc., Chicago, IL, USA). The Chi-square test was used to determine the potential relationship between SD and two variables of age and gender. A *p-value* less than 0.05 was considered statistically significant.

## Results and discussion

In the present study, the universal primers ITS4 and ITS5 were used to amplify the ITS5.8S rDNA region. A total of 23 isolates of *Malassezia* strains and 22 isolates of other fungal species were taken from 19 patients with SD (Table 1). Analysis of the relationship between SD and demographic characteristics did not show a significant association between SD and age and gender (*P*>0.07(. According to the data, the mean age of the participants was 30.4 years (Table 1). 

**Table 1:** Isolation of non-*Malassezia* and *Malassezia* clinical strains from patients with seborrheic dermatitis

	Fungal species	Number (%)	Gen bank accession numbers	Gender	Age (Year)
**Genus non-*Malassezia***	*Cryptococcus albidus var. albidus*	6 (13.3)	MN044934, MN044938	Male=8 Female=11	Range=17-48 Mean=30.4
			MN044935, MN044939		
			MN044936, MN044937		
	*Candida parapsilosis*	7 (15.5)	MN044943, MN044945		
			MN044946, MN044933		
			MN044944, MN080442		
			MN080443		
	*Penicillium polonicum*	3 (6.7)	MN044940, MN044941		
			MN044942		
	*Rhodotorula mucilaginosa*	6 (13.3)	MN044927, MN044931		
			MN044932, MN044928		
			MN044930, MN044929		
**Genus *Malassezia***	*M. furfur*	9 (20.01)	-		
	*M. globosa*	7 (15.5)	-		
	*M. sloofie*	3 (6.7)	-		
	*M. sympodialis*	3 (6.7)	-		
	*M. restricta*	1 (2.2)	-		

**Figure 1:**
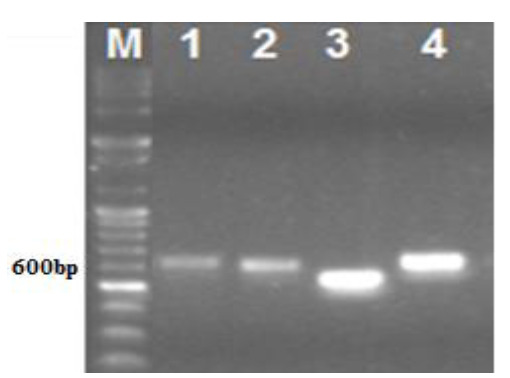
Gel electrophoresis of polymerase chain reaction products of isolated non- *Malassezia* species from SD scales on 1% agarose gel; lanes 1-4) *Cryptococcus albidus var. albidus, Candida parapsilosis*, *Rhodotorula mucilaginosa,* and *Penicillium polonicum*, lane M) 1-kb DNA ladder

The patients had no predisposing factor contributing to their infection. The fungal isolates detected in the samples included *Cryptococcus albidus var. albidus* (n=6,13.3%), *Candida parapsilosis* (n=7,15.5%), Rhodotorula mucilaginosa (n=6,13.3%), and *Penicillium polonicum* (n=3,6.7%; Table 1, Figure 1). These microorganisms rarely cause opportunistic fungal infections in immunocompromised hosts despite their common presence on the skin surface of the healthy subjects [ 3 ]. Nevertheless, in this study, *Candida parapsilosis* (15.5%; *P*˂0.001) was found to be the most common non-*Malassezia* species isolated from SD lesions.

*Candida parapsilosis*, as an opportunistic pathogen in human, can cause different infections, such as cerebritis, pneumonia, endocarditis, peritonitis, osteomyelitis, arthritis, and onychomycosis [ 18 , 19 ]. 

Recent evidence is suggestive of the associations between the increased resistance level of SD lesions in dogs and the co-colonization of two yeasts, namely *C. parapsilosis* and *M. pachydermatis* [ 12 ]. This can be explained by the growth of predominant strains by the first colonizing organism, which crowds out others in the same niche. Moreover, *C. parapsilosis* can produce more biofilms associated with *Malassezia* species. The amount of biofilm produced from the co-colonization of these yeasts was higher than that produced from single strains in vitro. This biofilm could exacerbate clinical symptoms by increasing the obstruction risk of sebaceous glands, leading to skin inflammation. It also serves as a drug sponge with antifungal sequestration abilities that can reduce azole activities against fungi [ 12 , 20 ]. The results of the present study regarding the fungal communities on the lesions of SD provided new evidence and confirmed previous findings. The current study is the first attempt targeting fungal microbiota in SD lesions in Iranian samples.

In conclusion, the results of the present study may provide basic information about the presence of a specific fungal population in SD lesions. However, our samples are too small to prove this claim. Therefore, further studies are recommended to investigate a larger sample size in order to evaluate the relationship between fungal species and SD.
